# Intrinsic Mechanisms Regulating Neuronal Migration in the Postnatal Brain

**DOI:** 10.3389/fncel.2020.620379

**Published:** 2021-01-13

**Authors:** Cedric Bressan, Armen Saghatelyan

**Affiliations:** ^1^CERVO Brain Research Center, Quebec City, QC, Canada; ^2^Department of Psychiatry and Neuroscience, Université Laval, Quebec City, QC, Canada

**Keywords:** neuronal migration, adult neurogenesis, olfactory bulb (OB), autophagy, intrinsic mechanisms, ATP/ADP, neurodegenerative disorders

## Abstract

Neuronal migration is a fundamental brain development process that allows cells to move from their birthplaces to their sites of integration. Although neuronal migration largely ceases during embryonic and early postnatal development, neuroblasts continue to be produced and to migrate to a few regions of the adult brain such as the dentate gyrus and the subventricular zone (SVZ). In the SVZ, a large number of neuroblasts migrate into the olfactory bulb (OB) along the rostral migratory stream (RMS). Neuroblasts migrate in chains in a tightly organized micro-environment composed of astrocytes that ensheath the chains of neuroblasts and regulate their migration; the blood vessels that are used by neuroblasts as a physical scaffold and a source of molecular factors; and axons that modulate neuronal migration. In addition to diverse sets of extrinsic micro-environmental cues, long-distance neuronal migration involves a number of intrinsic mechanisms, including membrane and cytoskeleton remodeling, Ca^2+^ signaling, mitochondria dynamics, energy consumption, and autophagy. All these mechanisms are required to cope with the different micro-environment signals and maintain cellular homeostasis in order to sustain the proper dynamics of migrating neuroblasts and their faithful arrival in the target regions. Neuroblasts in the postnatal brain not only migrate into the OB but may also deviate from their normal path to migrate to a site of injury induced by a stroke or by certain neurodegenerative disorders. In this review, we will focus on the intrinsic mechanisms that regulate long-distance neuroblast migration in the adult brain and on how these pathways may be modulated to control the recruitment of neuroblasts to damaged/diseased brain areas.

## Introduction

Neuroblasts are immature cells of neuronal lineage that are highly motile and migrate to target brain regions from their birthplaces to become neurons and integrate into neural networks. They are polarized cells, with a long leading and a short trailing process. During cell migration, neuroblasts do not migrate continuously and have saltatory movements with migratory periods intercalated by stationary phases (Wichterle et al., 1997; Komuro et al., 2001; Schaar and McConnell, 2005; Nam et al., 2007; Snapyan et al., 2009). These stationary phases are as important as the migratory periods and while the cell body is immobile during the stationary phases the leading process remains highly motile. Cell migration is associated with several morphological changes and re-arrangements of the cell body and processes, including the extension of the leading process, swelling formation in the leading process, centrosome movement (centrokinesis), and nucleus translocation (nucleokinesis) followed by the retraction of the trailing process (Lalli, 2014; Kaneko et al., 2017). The leading process is a very dynamic structure that allows the cells to sense their environment, determine the direction of migration, and attach to the extracellular matrix (ECM) to create traction forces (Martini et al., 2009) (Gardel et al., 2010). Swelling formation takes place in the proximal part of the leading process before nucleokinesis and is caused by the movement of the centrosome, Golgi apparatus, primary cilium, and mitochondria away from the nucleus (Bellion et al., 2005; Schaar and McConnell, 2005; Matsumoto et al., 2019). The nucleus, which is linked to the centrosome by microtubules, then moves forward along the microtubules due to the contraction of the cytoskeleton (Tsai and Gleeson, 2005).

**Figure 1:**
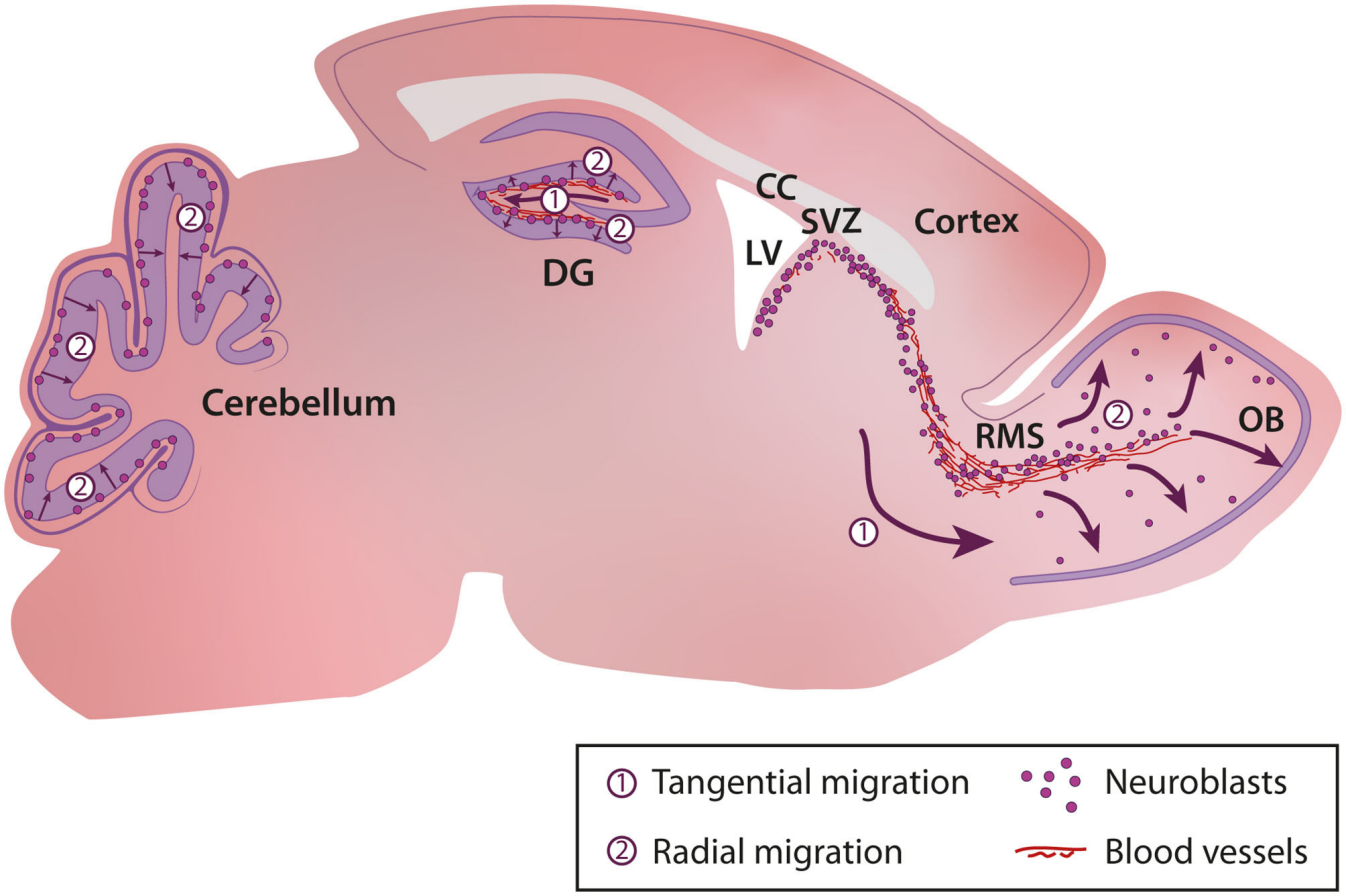
Cellular migration in the postnatal brain. In the postnatal brain migration occurs in the sub-granular zone (SGZ) of the dentate gyrus in the hippocampus, the rostral migratory stream and olfactory bulb, and the cerebellum. CC, Corpus Callosum; DG, Dentate Gyrus; LV, Lateral Ventricle; OB, Olfactory Bulb; RMS, Rostral Migratory Stream; SVZ, Subventricular Zone.

In the uninjured postnatal brain, neurogenesis and cell migration are largely restricted to a few specific regions such as the sub-granular zone (SGZ) of the dentate gyrus in the hippocampus (Altman and Das, 1965a,b), the subventricular zone (SVZ) bordering the lateral ventricle that provides cells for the olfactory bulb (OB) (Altman, 1969; Lois and Alvarez-Buylla, 1993, 1994), and the cerebellum (Altman and Das, 1965b) (Figure 1). During adulthood, stem cells (or B cells) in the SVZ give rise to transient amplifying cells (C cells) that in turn give rise to immature neuronal progenitors also known as neuroblasts (A cells) (Lois et al., 1996; Alvarez-Buylla et al., 2001). Neuroblasts, after a long migratory journey along the rostral migratory stream (RMS) integrate into the existing neuronal network in the OB (Lledo and Saghatelyan, 2005). Neuroblasts migrate tangentially in the RMS until they reach the OB, where they switch their mode of migration from tangential to radial (Lledo and Saghatelyan, 2005). In the hippocampus, neurogenesis and newborn neuron integration is restricted to the dentate gyrus (Abbott and Nigussie, 2020). As in the SVZ, progenitors in the SGZ migrate first tangentially away from their site of birth and then radially into the granule cell layer of the dentate gyrus (Sun et al., 2015). In the cerebellum, different waves of interneuron migration take place during the first three postnatal weeks. These interneurons, which originate from distinct primary germinal niches, migrate through the white matter or the external granular layer before reaching their final destination in the internal granular layer or molecular layer (Galas et al., 2017). Depending on their site of birth and their fate, distinct interneuronal progenitors exhibit different patterns of tangential and radial migration (Galas et al., 2017).

The characteristics of cell migration such as speed, distance of migration, tortuosity, and duration and periodicity of the migratory and stationary phases are variable and depend on cues in the surrounding micro-environment and the intrinsic properties of neuroblasts. For example, in the cerebellum, cells migrate faster in the middle of the external granular cell layer (EGL) than in superficial and deep layers of the EGL (Komuro and Rakic, 1995; Komuro et al., 2001). In the SVZ-OB pathway, the tangential migration of neuroblasts is slower in the SVZ than in the RMS (Nam et al., 2007) and is even slower in the core of the OB when the cells initiate their radial migration (David et al., 2013).

Neuroblasts migrate in a very complex micro-environment and are guided by different molecular cues, physical scaffolds, and electrical signals. In the present review, after a brief description of the different micro-environmental signals, we will focus on the intrinsic mechanisms that operate in neuroblasts and that enable them to cope with distinct micro-environmental signals in order to regulate neuronal migration in the SVZ-OB pathway.

### Molecular Cues

Neuronal migration relies on a large number of molecular cues that collectively regulate cell movement. Some of these factors regulate the pace and periodicity of neuronal migration, while others orchestrate the directionality and faithful arrival of neuroblasts in the OB. Neuroblasts express netrin receptors, neogenin, and deleted in colorectal carcinoma (DCC) and may react to netrin-1 expressed by mitral cells in the OB (Murase and Horwitz, 2002). Other factors secreted by the OB such as glial cell line-derived neurotrophic factor (GDNF) (Paratcha et al., 2006) and prokineticin 2 (Ng et al., 2005) may act as chemoattractants for neuroblast migration. The choroid plexus and septum secrete the repulsive factors Slit1 and Slit2 that may orient neuroblast migration away from the SVZ to the OB (Gotz and Stricker, 2006; Sawamoto et al., 2006). In the adult RMS, neuroblasts express Robo, the Slit receptor (Hu and Rutishauser, 1996; Wu et al., 1999) and may themselves release Slit to regulate migration in a cell autonomous manner (Nguyen-Ba-Charvet et al., 2004). Slit-ROBO signaling is also involved in astrocyte-neuroblast interactions in the RMS, indicating that the effect of Slit on neuroblast migration is not just limited to mechanisms to repel neuroblasts from the SVZ (Kaneko et al., 2010). Furthermore, it is not precisely known how chemoattractant and chemorepellent cues orient neuroblast migration in a complex and convoluted migratory stream that likely requires a multitude of different gradients acting in different directions (Gotz and Stricker, 2006; Snapyan et al., 2009). The faithful arrival of neuroblasts in the OB and their directionality of migration may also depend on the physical interactions of these migratory cells with surrounding cells such as astrocytes and blood vessels (Snapyan et al., 2009; Whitman et al., 2009; Kaneko et al., 2010) that topographically organize the migratory stream, as discussed below.

Neuronal migration is also regulated by a number of growth factors and neurotransmitters. Vasculature endothelial growth factor (VEGF) promotes neuronal migration in the RMS (Wittko et al., 2009). VEGF receptor 1 (VEGF-R1) is expressed by astrocytes. The downregulation of its expression leads to the accumulation of VEGF and increased phosphorylation of VEGFR-2 in neuroblasts (Wittko et al., 2009). Brain-derived neurotrophic factor (BDNF) also plays a role in migration by fostering the entry of neuroblasts into migratory phases (Snapyan et al., 2009). BDNF is secreted by the endothelial cells of blood vessels in the RMS, while its receptors TrkB and p75NTR are expressed by astrocytes and neuroblasts, respectively (Galvão et al., 2008; Snapyan et al., 2009). BDNF regulates neuroblasts migration via p75NTR expressed on neuroblasts, while neuroblasts via release of GABA induce insertion of high-affinity TrkB on the plasma membrane of astrocytes which in turn regulate extracellular levels of BDNF. This renders BDNF unavailable for p75NTR-expressing migrating cells and leads to their entry into the stationary phase, indicating that neuroblasts may regulate the periodicity of their own migration by dynamic regulation of BDNF level (Snapyan et al., 2009). GABA also controls neuroblast migration in a cell autonomous manner (Bolteus and Bordey, 2004; Snapyan et al., 2009). GABA affects the speed of neuroblast migration via Ca^2+^ activity and, independently, GABA-induced depolarization (Bolteus and Bordey, 2004). Neuroblasts also express glutamate receptors, and it has been shown that astrocyte-derived glutamate affects neuronal migration (Platel et al., 2008, 2010). Interestingly, both GABA and glutamate are released non-synaptically. Non-synaptic scaling of these factors in response to injury may adjust neuroblast production and migration (Platel et al., 2008). In addition to these non-synaptically released neurotransmitters, neuroblast migration in the adult RMS is also under the control of other neurotransmitters. The RMS is innervated by serotoninergic axons originating from raphe nuclei. The optogenetic stimulation of these axons increases the speed of migration via 5HT3A receptors on neuroblasts (García-González et al., 2017). Several other long-ranging axonal projections may impinge on the RMS, as they do in the SVZ (Hoglinger et al., 2004; Tong et al., 2014; Paul et al., 2017). The roles of other neurotransmitter systems in the regulation of neuronal migration remain to be investigated.

### Physical Cues

Neuroblast migration is regulated not only by secreted molecular cues but also by physical interactions with surrounding cells. The RMS has a highly organized shape along which neuroblasts leaving the SVZ form chains of migrating cells (Lois et al., 1996). In this context, cell-cell interactions and adhesion appear necessary to maintain neuroblast chain organization and promote normal migration. The polysialated neural cell adhesion molecule (PSA-NCAM) expressed by neuroblasts is also required to maintain chain migration in the RMS (Hu et al., 1996). The enzymatic removal of PSA or the genetic downregulation of NCAM leads to the disorganization of neuroblast chains in the RMS and their dispersion in surrounding regions (Chazal et al., 2000; Battista and Rutishauser, 2010). Chain formation is also regulated by the receptor tyrosine kinase ErbB4 as well as by their ligands (neuregulins) expressed in adjacent brain regions (Anton et al., 2004). Other receptor tyrosine kinases such as Eph receptors also regulate neuronal migration in the RMS. The infusion of ephrin-B2 into the lateral ventricle disrupts neuronal migration (Conover et al., 2000). Chain formation is also under the control of β1-integrin. A deficiency in β1-integrin signaling leads to chain disorganization and ectopic migration of neuroblasts to adjacent brain regions (Belvindrah et al., 2007; Fujioka et al., 2017). The RMS is a highly packed micro-environment, and neuroblasts migrating in chains use other neuroblasts in the chain as a scaffold for migration (Hikita et al., 2014). Morphological modifications in resting, non-migrating neuroblasts via Rac1-induced indentation of the cell body allows migrating neuroblasts to move forward (Hikita et al., 2014). The time-lapse imaging of neuroblasts in the organotypic slices has shown that when neuroblasts arrive into the OB and initiate their radial migration, they form a filopodium-like lateral protrusion on the proximal part of their leading process (Sawada et al., 2018). These protrusions are induced by PlexinD1 downregulation and local Rac1 activation, and are thought to be important for the termination of neuronal migration in the OB (Sawada et al., 2018).

In addition to neuroblast-neuroblast interactions, other cellular elements such blood vessels and astrocytes also play a role in neuronal migration. The RMS is characterized by a highly organized blood vessel architecture that topographically outlines the migratory stream and that is parallel to the migrating cells (Snapyan et al., 2009; Whitman et al., 2009). Neuroblasts use these blood vessels as a physical scaffold for migration, and the cell body or leading process remains close to them during the entire process of cell migration (Bovetti et al., 2007b; Snapyan et al., 2009; Whitman et al., 2009). The parallel organization of blood vessels is guided by astrocyte-derived VEGF during early postnatal development (Bozoyan et al., 2012). Vasculature-mediated neuronal migration appears as the predominant mode of cell navigation in the postnatal brain and is involved in the migration of endogenous and grafted neuronal progenitors under homeostatic (Betarbet et al., 1996; Honda et al., 2007; Le Magueresse et al., 2012; Sun et al., 2015; Lassiter et al., 2016) and pathological conditions (Ohab et al., 2006; Yamashita et al., 2006; Thored et al., 2007; Kojima et al., 2010; Grade et al., 2013; Fujioka et al., 2017). The interaction with blood vessels is regulated by β1-integrins expressed by migrating neuroblasts (Fujioka et al., 2017). In addition, endothelial cells secrete BDNF to orchestrate the pace and periodicity of neuronal migration (Snapyan et al., 2009; Grade et al., 2013). β1-integrins on neuroblasts, by binding to laminin in the RMS (Belvindrah et al., 2007) and post-stroke striatum (Fujioka et al., 2017), facilitate neuronal migration. Laminin is an important component of the physical scaffold during neuronal migration and artificial scaffolds covered with laminin enhance cell migration (Ajioka et al., 2015; Fujioka et al., 2017). Similarly, BDNF, which is involved in vasculature-guided neuronal migration in physiological and pathological conditions (Snapyan et al., 2009; Grade et al., 2013), promotes neuronal migration when present in artificial nanofibrous scaffolds and redirects neuroblast migration to ectopic brain regions (Fon et al., 2014).

Neuroblasts are ensheathed by a glial tube forming a tunnel that confines neuroblasts migration to the RMS and, at the same time, modulates the migratory behavior of cells through the release of different molecules or neuroblast-astrocyte physical interactions. The disorganization of the glial tube or an inefficient interaction between astrocytes and neuroblasts leads to drastic changes in neuroblast migration (Chazal et al., 2000; Anton et al., 2004; Belvindrah et al., 2007; Kaneko et al., 2010). RMS astrocytes are molecularly distinct and play an important supporting role in neuronal migration. SVZ explants co-cultured on RMS-derived astrocytes exhibit more migration than explants cultured on cortical astrocytes (García-Marqués et al., 2010). These differences are determined by astrocyte-mediated non-soluble factors (García-Marqués et al., 2010). The organization of astrocytes is also an important factor in preserving an efficient migratory path. Neuroblasts themselves prevent astrocytic processes from invading the RMS by secreting Slit1, which acts on astrocytic Robo receptors to repel astrocytic processes out of the migratory path (Kaneko et al., 2010). The Slit-Robo-mediated neuroblast-astrocyte interaction is also involved in neuronal migration during injury and allows neuroblasts to migrate through reactive astrocytes close to the lesion site (Kaneko et al., 2018). While all these studies have revealed several levels of physical contacts and intercellular interactions that regulate neuronal migration, the repertoire of mechanoreceptors on neuroblasts and the role of mechanical forces in their migration is unclear. This is particularly relevant since the RMS is characterized by a very high cell density and, consequently, mechanical forces may determine the efficiency of neuronal migration.

In line with this, adhesion to the ECM is required for migrating cells to generate mechanical forces for movement through adhesive contacts mediated by integrin-based focal adhesions (Gardel et al., 2010). ECM metalloproteases (MMP) play important roles in cell migration. Neuroblasts release MMP5, which is required to maintain migration (Lee et al., 2006; Bovetti et al., 2007a). The outer border of the RMS contains secretagogin-positive neurons that support migration by releasing MMP2 (Hanics et al., 2017). The ECM plays also an important role in neuroblast detachment and radial migration following the arrival of neuronal precursors in the OB. Reelin expressed by mitral cells in the OB induces the detachment of neuroblasts from chains (Hack et al., 2002) while tenascin-R, which is expressed in the OB and but not in the RMS, mediates radial migration and the activity-dependent recruitment of neuroblasts into the OB (Saghatelyan et al., 2004).

### Galvonotaxis

Together with molecular cues and physical factors, electrical currents appear to be another component of cell guidance. Several studies have shown that the application of an electric field increases neurogenesis (Stone et al., 2011; Sefton et al., 2020) and drives neuroblasts toward the cathode in cell cultures, SVZ explants, and acute brain slices (Babona-Pilipos et al., 2011; Cao et al., 2013). Human neuronal progenitor cells (hNPCs) also migrate to the cathode in response to an electric field (Feng et al., 2012). In the adult mouse brain, the RMS exhibits an electric potential gradient of ~3 mV/mm from the SVZ to the OB (Cao et al., 2013). The application of a higher electric field gradient against the natural RMS gradient redirects the migration of grafted NPCs (Feng et al., 2017; Iwasa et al., 2019). These galvanotactic effects seem to be mediated by P2Y1 purinergic receptors and are accompanied by Ca^2+^ fluctuations (Cao et al., 2013; Babona-Pilipos et al., 2018). The downregulation of P2Y1 receptors in neuroblasts results in a loss of directionality of migration in response to an electric field (Cao et al., 2013). It is not known whether galvanotaxis plays a role in the migration in other brain regions when the migration distance is more limited or how these electrical gradients are established.

All these extrinsic factors are constantly sensed by neuroblasts during migration and have to be integrated and decoded at the cellular level. Once properly decoded, they lead to the morphological changes required for cell movement. One of the important sensors that links different extrinsic factors with intracellular pathways is primary cilium, a centrosome-associated structure present at the surface of all eukaryotic cells. The spatio-temporal dynamics of primary cilia is associated with saltatory movement of neuroblasts and tangential migration depending on the activation of cAMP-PKA pathways (Matsumoto et al., 2019; Stoufflet et al., 2020). In this review, we will focus on some of the intrinsic pathways that regulate neuronal motility and migration in response to micro-environment cues.

## Intrinsic Factors That Allow Neuronal Migration

### Cytoskeleton Remodeling

Cell movement is supported by spatially and temporally organized rearrangements of the cytoskeleton that regulate the different steps of migration. These cytoskeleton rearrangements occur in response to extracellular signals and are mediated by intracellular signaling pathways (Ayala et al., 2007). The cytoskeleton is composed of actin filaments, microtubules, and intermediate filaments (IF) that control different aspects of cellular organization and motility.

#### Actin Filaments

Actin filament assembly and disassembly is a key mechanism that orchestrates cell movement. The suppression of actin polymerization leads to a complete failure of neuronal migration (Rivas and Hatten, 1995; Fox et al., 1998). Actin filaments (also called F-actin) are composed of double helical polymers of actin subunits. The filaments are polarized, with their plus (barbed)-end toward the leading edge of the cell (Lehtimäki et al., 2017). Actin assembly can occur either via elongation or branching. Actin filaments are enriched at the front of the cells (Wilson et al., 2013; Diz-Muñoz et al., 2016; Bisaria et al., 2020). The outer layer of the actin network, also called membrane proximal F-actin, links actin to the cell membrane by a linker protein and exhibits a reverse back-to-front gradient, with the lowest density at the front of the cells. This results in low attachment to the cell membrane and facilitates membrane protrusion (Bisaria et al., 2020).

The movement of the actin network is regulated by the actin-based motor proteins such as non-muscle myosin II which forms a network referred as actomyosin (Bellion et al., 2005; Schaar and McConnell, 2005). Actomyosin fibers form bundles or stress fibers in cells that are anchored to the ECM by focal adhesions (FA) and that produce the contractile forces (Naumanen et al., 2008; Tojkander et al., 2012). Myosin IIA and IIB are predominantly expressed in migrating cells in the CNS. A point mutation in one of these myosins can result in impaired migration *in vivo* (Ma et al., 2004; Dulyaninova et al., 2007). Myosin IIA and IIB are differentially distributed at the leading edge and at the rear of migrating cells, respectively (Kolega, 1998). Myosin II is mainly controlled by myosin light chain (MLC) phosphorylation. It is phosphorylated by MLC kinase and Rho-Kinase (Hartshorne et al., 1998; Kamm and Stull, 2001). Actomyosin contraction at the rear of the cell creates the driving force that pushes the nucleus toward the leading process (Bellion et al., 2005; Martini and Valdeolmillos, 2010) and helps cells detach from extracellular adhesions and retract the trailing process (Schaar and McConnell, 2005).

The regulation of actin and myosin is dependent on Rho GTPases. Ras homologous member A (RhoA), Ras-related C3 botulinum toxin substrate 1 (Rac1), and cell division cycle 42 (Cdc42) are particularly important in controlling actin network remodeling during cell migration (Luo, 2000; Ayala et al., 2007). The expression of constitutively active RhoA, Rac1 dominant negative (DN), and Cdc42 DN inhibit cell migration in SVZ explants (Wong et al., 2001). These Rho GTPases are modulated by two classes of antagonist proteins, that is, GTPase-activating proteins (GAPs) and guanine nucleotide exchange factors (GEFs) (Luo, 2000). A number of extrinsic molecular cues regulate neuronal migration by affecting these pathways. For example, the Slit-Robo pathway depends on a direct interaction between Robo1 and a specific GAP, resulting in the direct or indirect modulation of Rho GTPases and the inactivation of Cdc42 (Wong et al., 2001). Rac plays an important role in membrane protrusions and is enriched at the front of migrating cells (Yang et al., 2016; Bisaria et al., 2020). Rac1 activation is also implicated in the PlexinD1-regulated formation of filopodium-like lateral protrusion on the leading process of radially migrating cells in the OB, which is associated with the termination of radial migration (Sawada et al., 2018). Rac and Cdc42 have a common effector, p-21activated-kinase (Pak) (Ayala et al., 2007). Pak activation leads to a molecular cascade resulting in the inhibition of cofilin, an actin depolymerization factor (Arber et al., 1998; Yang et al., 1998). Pak also decreases the activity of myosin-light chain kinase (MLCK) that phosphorylate myosin II (Sanders et al., 1999). Cofilin is an important regulator of the front-to-rear membrane proximal F-actin gradient (Bisaria et al., 2020). MLCK regulates membrane protrusions and focal adhesion assembly at the edge of the membrane protrusion (Totsukawa et al., 2004) and is involved in the formation of the fibronectin-integrin-cytoskeleton complex that regulates the membrane F-actin skeleton (Chen et al., 2014). In RMS neuroblasts, shootin1b couples F-actin retrograde flow and adhesive substrates as a clutch molecule, which creates a traction force that promotes leading process extension and soma translocation (Minegishi et al., 2018). Cdc42 also plays an important role in *de novo* F-actin assembly by indirectly activating the actin-related protein 2/3 (Arp2/3) complex (Rohatgi et al., 1999). This Arp2/3 complex regulates the formation of branched actin filaments by directly interacting with existing active filaments to rapidly create new ones (Mullins et al., 1998; Smith et al., 2013). Lastly, RhoA profilin regulates actin polymerization, profilin-1 promotes membrane protrusion and cell motility, while profilin-2 inhibits these processes (Mouneimne et al., 2012). Interestingly, GTPases have a specific spatiotemporal pattern of activation with RhoA activation at the front of the cell followed by activation of Cdc42 and Rac1 on the edge of the cell (Machacek et al., 2009; Tkachenko et al., 2011). RhoA modulation also contributes to the saltatory movement of neuroblasts by controlling swelling formation (Shinohara et al., 2012; Ota et al., 2014).

#### Microtubules

Microtubules are the second cytoskeleton component involved in regulating cell migration. Microtubules are hollow filaments composed of α-tubulin and β-tubulin heterodimers (Garcin and Straube, 2019). Like actin filaments, they are polarized, with a plus end with exposed β-tubulin and a minus end with exposed α-tubulin (Garcin and Straube, 2019). Microtubule functions are mediated by microtubule-associated proteins (MAPs) and the motor proteins, kinesin and dynein (Dehmelt and Halpain, 2004). Microtubule organizing centers (MTOCs), generally the centrosome, are the start point for microtubule assembly (Etienne-Manneville, 2013). A recent study identified AKNA, a centrosome protein, as a major player in centrosomal microtubule organization in neural stem cells (NSCs) in the developing cortex (Camargo Ortega et al., 2019). AKNA promotes the nucleation and growth of centrosomal microtubules in specific subpopulations in the SVZ, which leads to the delamination and formation of the SVZ (Camargo Ortega et al., 2019). AKNA is also involved in the epithelial-mesenchymal transition (EMT) in other epithelial cells and neuronal migration in the developing cortex (Camargo Ortega et al., 2019). The end-binding protein EB1, a MAP recruited to microtubules by AKNA (Camargo Ortega et al., 2019), is involved in cell protrusion (Gierke and Wittmann, 2012; Jayatilaka et al., 2018).

Microtubule filament polarity allows molecular motors and cargo, including membrane vesicles, signaling molecules, RNAs, and cytoskeletal compounds, to move directionally along the microtubules (Etienne-Manneville, 2013; Garcin and Straube, 2019). Microtubules are able to act on actin polymerization by delivering GEFs to protrusion sites to modulate Rac activity (van Horck et al., 2001; Rooney et al., 2010). During cell migration, STIM1, an important controller of ER Ca^2+^ store levels, is transported along the microtubule plus end at the front of cells (Tsai et al., 2014). Mitochondria are another important cargo transported by microtubules. Microtubules, through the interaction of kinesin with mitochondrial Rho-GTPases1 (MIRO1), carry mitochondria to the leading edge of the cell (Guo et al., 2005; Glater et al., 2006; Schuler et al., 2017). Microtubules are also important in regulating focal adhesions by controlling the delivery of adenomatous polyposis coli (APC) to the plus end of the microtubule (Mimori-Kiyosue et al., 2000). APC is involved in regulating focal adhesions in migrating cells (Matsumoto et al., 2010). Microtubules also deliver mitogen-activated protein kinase 4 (MAP4K4) to focal adhesion complexes to promote their disassembly (Yue et al., 2014).

Altogether, these findings indicate that the polarized cytoskeleton in migrating cells acts as a dynamic scaffold and contributes to intracellular organization by regulating the distribution and targeting of different proteins and organelles.

#### Intermediate Filaments

Intermediate filaments (IFs) are a large family of proteins involved in different cellular functions. These proteins are grouped into different subsets such as type III, which includes vimentin and glial fibrillary acidic protein (GFAP), type V, which includes laminin family proteins, and type VI, which includes nestin (Bernal and Arranz, 2018; Bott and Winckler, 2020). IFs are composed of coiled-coil dimers that are assembled into filaments with a slow disassembly rate (Bott and Winckler, 2020). IFs are required to maintain migration and astrocytes lacking IF vimentin or GFAP exhibit impaired mobility (Lepekhin et al., 2001).

IF crosstalk with microtubules and the actin regulatory pathway. Actin-associated kinases such as ROCK and Pak are involved in the regulation of IF microtubule-dependent transport (Robert et al., 2014). IFs can also modulate actin formation just as keratin regulates cell contractility during collective cell migration by inhibiting Rac1 activation at the rear of the cell and acting on F-actin formation (Sonavane et al., 2017). Similarly, vimentin can regulate Rho activity to inhibit actin stress fiber assembly (Jiu et al., 2017). IF vimentin can also directly interact with F-actin by the tail domain and enhance the stiffness of the F-actin/vimentin network (Esue et al., 2006). Interestingly, in dense environments, the increase in stiffness due to IF vimentin confers an advantage on cells by sustaining migration (Messica et al., 2017). In NSCs, the suppression of nestin inhibits migration and contraction *in vitro* by affecting myosin regulatory light chain and myosin light chain kinase (Yan et al., 2016). IF vimentin is required to maintain microtubule-induced polarity during retinal pigment epithelial cell migration by templating and stabilizing microtubule organization (Gan et al., 2016).

IFs also control the distribution and dynamics of FA and FA-mediated traction forces during the collective migration of astrocytes during development (De Pascalis et al., 2018). Protein levels of vimentin regulate FA size (Terriac et al., 2017). By controlling FA dynamics, IFs can modulate the speed of cell migration (Terriac et al., 2017; De Pascalis et al., 2018). In fibroblasts, the suppression of vimentin reduces FA turnover and impairs migration directionality (Gregor et al., 2014).

IFs are expressed in a cell-lineage restricted manner (Bott and Winckler, 2020). In the CNS, GFAP and vimentin are expressed by astrocytes (Lepekhin et al., 2001; De Pascalis et al., 2018) while GFAP and nestin are expressed by stem cells (Lendahl et al., 1990; Doetsch et al., 1999; Codega et al., 2014). Nestin is involved in the migration of a immortalized mouse neural progenitor cell line (Yan et al., 2016). Little is known about the expression profile of neuroblast IFs. The exact role played by IFs in these migratory cells needs to be explored.

### Ca^2+^ Signaling

Ca^2+^ signaling is involved in the regulation of a variety of biological processes ranging from cell proliferation to cell death (Kawamoto et al., 2012). In migrating cells, spontaneous Ca^2+^ activity is required to maintain actin contraction leading to nucleokinesis (Martini and Valdeolmillos, 2010). Spontaneous and depolarization-induced Ca^2+^ transients have been observed in both tangentially and radially migrating neuroblasts in the SVZ-OB pathway (Darcy and Isaacson, 2009; García-González et al., 2017). Serotonin receptor 3A (5HT3A)-mediated Ca^2+^ influx is required to sustain the speed and directionality of neuroblasts that are tangentially migrating toward the OB (García-González et al., 2017). On the other hand, L-type Ca^2+^ channels mediate spontaneous and depolarization-induced Ca^2+^ signaling in radially migrating neuroblasts, but their inhibition does not affect the rate of migration (Darcy and Isaacson, 2009).

A number of micro-environmental signals may affect Ca^2+^ dynamics in migrating cells through G protein couple receptors (GPCRs) or by tyrosine receptor kinase (TRK) activation. These receptors may activate phospholipase C (PLC), which cleaves phosphatidylinositol 4,5 bisphosphate (PIP2) into 1,4,5-inositol triphosphate (IP3) and diacyl-glycerol (DAG) (Clapham, 2007). IP3 binds to the IP3 receptor at the surface of the endoplasmic reticulum (ER), resulting in a local or global rise in cytoplasmic Ca^2+^. The plasma membrane ER Ca^2+^ ATPase then pumps Ca^2+^ ions back to the extracellular space (Clapham, 2007). During cell migration, the spatiotemporal regulation of Ca^2+^ concentrations and dynamics are highly regulated. Migrating cells display a Ca^2+^ gradient from rear to front, which may be required for directional migration (Brundage et al., 1991; Gilbert et al., 1994). This rear-to-front Ca^2+^ gradient is determined by the polarized distribution of TKR, PLC, DAG, plasma membrane Ca^2+^ pumps, and STIM1 (Tsai et al., 2014).

Transient intracellular Ca^2+^ fluctuations that lead to the retraction of the rear of the cell have been observed in migrating cells (Lee et al., 1999). However, Ca^2+^ transients are larger in protrusions and at the front of cells (Blaser et al., 2006). Ca^2+^ microdomains at the front of cells play a role in steering directional migration (Wei et al., 2009, 2010) and are mediated by stretch-activated cation channels (TRPM7) (Wei et al., 2009). Local Ca^2+^ transients at the front of human endothelial cells activate MLCK and myosin II, allowing the contraction of actin filaments locally (Tsai and Meyer, 2012). These contractions result in the cycles of retraction of lamellipodia and the strengthening of paxillin-based focal adhesions (Tsai and Meyer, 2012). Extracellular Ca^2+^ influxes also maintain PI3K activity, which is proposed to be a major signaling hub in migrating cells (Khodosevich and Monyer, 2011) and, at the same time, promote F-actin polymerization and trigger the recruitment of Ca^2+^-sensitive enzyme protein kinase Cα (PKCα) to the plasma membrane of the leading edge of migrating cells (Evans and Falke, 2007). Ca^2+^ transients also enable the translocation of Rac1 to the plasma membrane, leading to the formation of lamellipodia (Price et al., 2003). Although local Ca^2+^ fluctuations are more frequent at the front of the migrating cells (Tsai et al., 2014), L-type voltage-dependent Ca^2+^ channels mediate Ca^2+^ sparklets at the rear of the cells, which plays a role in maintaining front-to-rear Ca^2+^ gradients (Kim et al., 2016). Ca^2+^ signaling also modulates mitochondria mobility and increases their availability at sites of high energy demand by uncoupling MIRO1 and kynesin (Saotome et al., 2008; Macaskill et al., 2009; Wang and Schwarz, 2009).

### Mitochondria Dynamics

Oxidative phosphorylation in mitochondria is the main source of ATP production in cells. Mitochondria are extremely dynamic organelles and continuously change their shape by undergoing fission (fragmentation) and fusion (elongation) (Bertholet et al., 2016). Mitochondria are transported along microtubules to sites of high energy demand (Cai et al., 2011; Cunniff et al., 2016). In migrating cells, mitochondria are organized in a radial distribution, with the majority positioned close to the nucleus (Schuler et al., 2017). During chemotaxis migration, mitochondria are positioned preferentially at the anterior part of the cell between the nucleus and the leading edge (Desai et al., 2013). In cellular protrusions, AMPK regulates mitochondria trafficking in order to drive local ATP production (Cunniff et al., 2016).

While the role of mitochondria dynamics in neuroblast migration in the adult brain remains to be investigated, several studies have shown that mitochondria are important for cell motility during cancer cell migration (Zhao et al., 2013; LeBleu et al., 2014; Cunniff et al., 2016; Vyas et al., 2016; Srinivasan et al., 2017; Sun et al., 2018a,b; Denisenko et al., 2019). In cancer cells, mitochondrial fission promotes focal adhesion dynamics and lamellipodia formation, which leads to increased migration (Sun et al., 2018a). Increases in mitochondrial DNA enhance metastasis by increasing oxidative phosphorylation and ATP production (LeBleu et al., 2014; Sun et al., 2018b). Levels of dynamin-related protein 1 (DRP1), a GTP enzyme molecule that is a key protein of mitochondrial fission, increase in carcinoma (Sun et al., 2018a). The downregulation of this enzyme leads to a decrease in lamellipodia formation by inducing F-actin remodeling (Zhao et al., 2013; Sun et al., 2018a; Ponte et al., 2020).

The mitochondrial electron transport chain produces reactive oxygen species (ROS) during the ATP production process (Wang et al., 2011) together with NADPH oxidase (Hurd et al., 2012). ROS may impact several intracellular pathways involved in migration, including actin cytoskeleton dynamics, cell adhesion, and MMPs, among others (Sena and Chandel, 2012; Tochhawng et al., 2013; Vyas et al., 2016). Increased ROS signaling in the cell supports cancer cell migration (Tochhawng et al., 2013; Kumari et al., 2018). ROS are able to activate the PI3K pathway by inactivating the phosphate and tensin homolog (PTEN), an inhibitor of PI3K activity (Lee et al., 2002; Sullivan and Chandel, 2014; Kumari et al., 2018). The role of ROS signaling in adult neuroblast migration remains to be investigated.

### Energy Consumption

Cell migration is a high energy process and is associated with major dynamic modifications in ATP levels. Cells require increased oxidative phosphorylation to migrate (LeBleu et al., 2014) and control the ATP/ADP ratio in order to sustain a normal rate of migration (Zhou et al., 2014; Zanotelli et al., 2018). In migrating cells, the ATP/ADP ratio has a rear-to-front gradient, with the lowest ratio at the leading edge of the cell despite higher ATP production compared to the cell body (Cunniff et al., 2016; Schuler et al., 2017). This ATP/ADP gradient is a sign of high energy consumption at the leading edge of the cell (Cunniff et al., 2016; Schuler et al., 2017).

**Figure 2:**
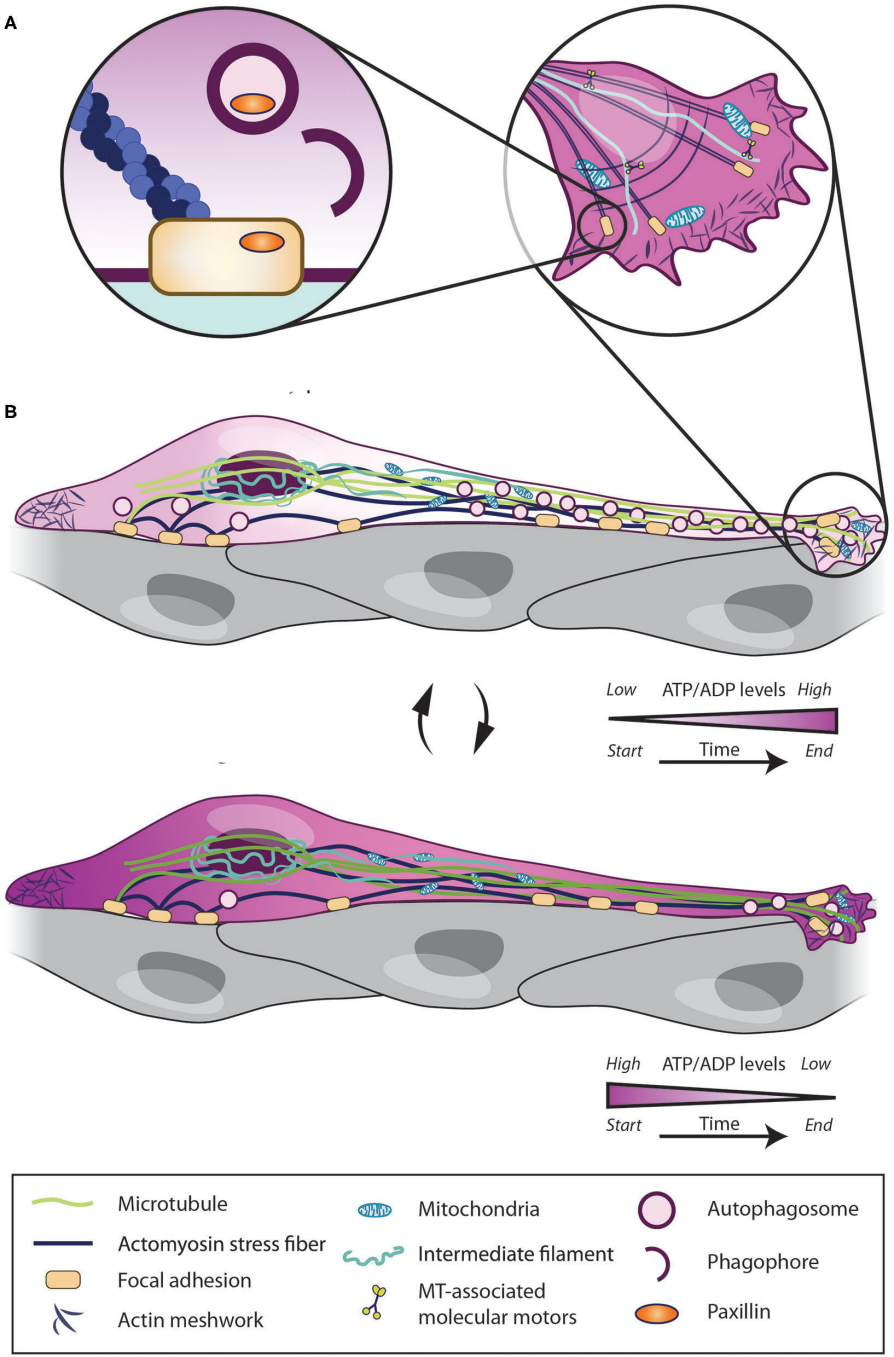
Intrinsic mechanisms regulating migratory and stationary phases of neuroblasts. Cell migration depends on intrinsic pathways such as Ca^2+^ signaling, cytoskeleton remodeling, mitochondria dynamics, energy consumption and autophagy. Neuroblasts use their stationary periods **(A)** to enhance their ATP/ADP levels and to recycle focal adhesion by autophagic process and enter into migratory phase. Once in migratory phases **(B)**, the intracellular ATP level rapidly decreases. When ATP level is too low cells again enter into stationary phases and start restoring the ATP/ADP levels.

The major sensor of the ATP/ADP ratio in cells is AMP activated kinase (AMPK), which is phosphorylated when the ATP/ADP ratio decreases (Amato and Man, 2011; Hardie et al., 2012). In migrating cells, AMPK activity increases at the leading edge of cells, which leads to mitochondria recruitment (Cunniff et al., 2016). AMPK activity is also involved in the phosphorylation of the microtubule-associated CLIP-70 protein, which is required for microtubule dynamics and, consequently, cell migration (Nakano et al., 2010). At the same time, however, AMPK activation can also block migration by suppressing the activity of Rac1 (Yan et al., 2015), an important Rho-GTPase involved in chain migration (Hikita et al., 2014). These findings indicate that context-dependent fine tuning of AMPK activity may be required to determine the efficiency of cell migration. Indeed, we recently showed that the dynamic modulation of the ATP/ADP ratio is required to determine the pace and periodicity of cell migration (Bressan et al., 2020). We used a ratiometiric ATP/ADP sensor to show that the migratory phases of tangentially migrating cells in the RMS are associated with high energy consumption and a gradual decrease in ATP levels. It is conceivable that, because of this drop in ATP levels, cells enter the stationary phase where ATP/ADP levels can recover (Bressan et al., 2020) (Figure 2). Interestingly, these changes in ATP/ADP levels in migrating cells are dynamically linked to autophagy, another major intracellular pathway, that maintains cellular homeostasis and sustains the pace and periodicity of neuronal migration in the postnatal and adult brain (Bressan et al., 2020).

### Autophagy

Autophagy is an evolutionarily conserved pathway first discovered in yeast. Autophagy allow cells to degrade and recycle proteins and organelles (Galluzzi et al., 2017). Autophagy can be either non-selective (macroautophagy) or highly selective by sequestering specific organelles (mitophagy, ribophagy, lipophagy, pexophagy, and others) (Farré and Subramani, 2016). Autophagy occurs in two steps. The first involves a formation of double-membrane vesicle called an autophagosome that encapsulates the part of the cytoplasm to be degraded. The vesicle then fuses with a lysosome to form an autolysosome, which degrades proteins and damaged organelles by the lysosomal machinery (Yang and Klionsky, 2010). Autophagy activation is mediated by two major upstream regulators and by different protein complexes that can be divided into six groups (Mizushima et al., 2011; Noda and Inagaki, 2015; Hurley and Young, 2017). Briefly the main regulators of autophagy, AMPK and mTOR, modulate the activity of the Atg1/ULK complex (Atg1, Atg13, Atg17, Atg29, and Atg31 in yeast and ULK1/2, ATG13, ATG101, and FIP200 in mammalian cells) (Lee et al., 2010; Egan et al., 2011; Kim et al., 2011; Shang et al., 2011). The kinase activity of this complex activates downstream effectors and recruits Atg9-containing vesicles to from an isolation membrane (or phagophore). The two ubiquitin-like conjugation systems then work together to link Atg8 (LC3 or LC3-I in mammals) with phosphatidylethanolamine (LC3-PE is also called LC3-II). In parallel, a class III PI3K complex, including vacuolar protein sorting (Vps) Vps34p-Vps15p, produces phosphatidylinositol 3-phosphate (PI3P), which is required for proper autophagosome formation (Mizushima et al., 2010, 2011; Yang and Klionsky, 2010; Noda and Inagaki, 2015; Antonioli et al., 2017; Graef, 2020). Lipidated LC3 is required for the closure of autophagosomes and for the docking of specific cargo receptors such as p62 and paxillin (Sharifi et al., 2016; Mizushima, 2020).

A number of studies have shown that autophagy is involved in the regulation of cell migration (Peng et al., 2012; Tuloup-Minguez et al., 2013; Lock et al., 2014; Hu et al., 2015; Li et al., 2015, 2019; Kenific et al., 2016; Sharifi et al., 2016; Petri et al., 2017; Gstrein et al., 2018; Bressan et al., 2020). The involvement of autophagy in cell migration has been demonstrated in cancer cells, cell lines and, more recently, in the CNS. For example, in HeLa cells, siRNA against Atg7 induced a migration deficit in a wound-healing migration essay (Tuloup-Minguez et al., 2013). In murine embryonic fibroblasts, Atg5 knock-out also produced a migration deficit in the same wound-healing assay (Li et al., 2015). The activation of mTOR signaling pathways in rat pulmonary arterial smooth muscle cells inhibits autophagy and decreases the migration of these cells in a hypoxic environment (Zhang et al., 2014). Some studies have also shown that autophagy activation in *in vitro* migration systems can have a negative effect (Tuloup-Minguez et al., 2013). As autophagy helps cells adapt to the extracellular environment, especially in hypoxia and normoxia, the effect of autophagy on cellular homeostasis and migration can be context dependent (Hu et al., 2015; Li et al., 2015).

In the developing brain, the Vps15 mutation in mice leads to cortical neuronal migration defects and, in humans, causes neurodevelopmental disorders (Gstrein et al., 2018). Similarly, suppression of Vps18, a central subunit of the Vps-C complex which regulates the vesicles transport to lysosome, impairs neuronal migration and induces neurodegeneration by impacting several vesicle transport pathways to the lysosome, including autophagy (Peng et al., 2012). In embryonic cortical cells, the ectopic expression of an autism-related FOXP1 mutation also leads to a concomitant migratory delay and autophagy activation (Li et al., 2019). The suppression of autophagy regulation by the knockdown of miRNA let-7 decreases the radial migration of newborn neurons in the OB while overexpressing beclin-1 and TFEB restores migratory behavior (Petri et al., 2017). We recently used pharmacological, genetic mouse model, and CRISPR-Cas9 approaches to perturb the expression of several autophagy genes, including Atg5, Atg12, Ulk1, and Ulk2 in tangentially migrating neuroblasts and studied the role played by this catabolic pathway in cell migration (Bressan et al., 2020). Autophagy impairment led to decreased migration of tangentially migrating neuroblasts because of the longer duration of the stationary phases (Bressan et al., 2020). This in turn induced an accumulation of neuroblasts in the SVZ *in vivo* and fewer cells in the core of the OB (Bressan et al., 2020).

Autophagy may modify neuronal migration by affecting several molecular cascades. Autophagy promotes cell migration by recycling focal adhesions via the autophagy cargo receptor NBR1 (Kenific et al., 2016). NRB1 is a signal transduction adaptor with an LC3-interacting domain (LIR) that is able to bind ubiquitin and LC3 to mediate selective autophagic degradation of ubiquitinated targets (Rogov et al., 2014). LC3 is also able to interact directly with paxillin through an LIR motif of paxillin (Sharifi et al., 2016). In the RMS, Atg5 cKO neuroblasts accumulate paxillin in the distal part of the leading process (Bressan et al., 2020). In migrating cancer cells, ULK1-FIP200 controls cell tumor mobility by activating AMPK and promoting the inhibition of the focal adhesion kinase, which immobilizes cells under low ATP conditions (Caino et al., 2013). Autophagy is also involved in the release of pro-migratory interleukin 6 and MMP2 (Lock et al., 2014). The Vps15 part of the class III PI3K complex is also involved in the stabilization of cytoskeleton dynamics by decreasing the activity of Pak1, an effector of Rac1 and Cdc42 (Gstrein et al., 2018). We recently showed that the dynamic interplay between ATP/ADP levels and autophagy is required to sustain neuronal migration in the postnatal and adult brain (Bressan and Saghatelyan, 2020; Bressan et al., 2020). A decrease in ATP levels during the migratory phase causes cells to enter into the stationary period and activates AMPK. This in turn induces autophagy, which is also associated with a concomitant increase in ATP levels (Bressan et al., 2020) (Figure 2). Blocking AMPK reduces the migration of wild-type cells but not of autophagy-deficient cells, providing a mechanistic link between these two processes (Bressan et al., 2020). Interestingly, autophagy is dynamically modulated by several migration-promoting or inhibiting molecular cues and is required to cope with the dynamics of cell migration, indicating that autophagy plays an important homeostatic role and adapts the intracellular recycling system in response to different micro-environmental cues (Bressan et al., 2020).

## Neuronal Migration in Human Neurodegenerative Disorders

Although adult neurogenesis occurs throughout the lifespan of rodents, neurogenesis in humans has recently come into question (Sorrells et al., 2018). It has been generally accepted that neuronal production persists in the adult human DG (Eriksson et al., 1998; Palmer et al., 2000; Spalding et al., 2013; Ernst et al., 2014), but the paper by Sorrells et al. indicates that human neurogenesis drops sharply after early brain development and is largely lacking in the adult brain (Sorrells et al., 2018). At the same time, another paper reported contradictory results (Boldrini et al., 2018), and recent studies have confirmed the presence of newborn cells in the adult human brain (Moreno-Jimenez et al., 2019; Tobin et al., 2019). The possible reasons for these discrepancies have been extensively discussed (Kempermann et al., 2018; Paredes et al., 2018; Snyder, 2018). For example, methodological issues related to delays in the fixation of postmortem samples (Kempermann et al., 2018; Paredes et al., 2018), the duration of the fixation period affecting immunohistological detection (Moreno-Jimenez et al., 2019; Flor-García et al., 2020) and the use of immunohistological labeling as the only method to detect adult neurogenesis (Kempermann et al., 2018; Paredes et al., 2018; Snyder, 2018) have been discussed. However, it has also been proposed that newborn neurons in adult primates and humans may retain their plastic properties for a longer time and may play a distinct functional role depending on their developmental stage despite their low numbers (Snyder, 2019). The advent of new imaging and gene sequencing technologies may help to address these issues and advance our knowledge.

The existence of the RMS in human adulthood has been also debated (Curtis et al., 2007; Sanai et al., 2007). It was later found that the infant SVZ-RMS contains a large number of migrating neurons, which decrease markedly with brain development (Sanai et al., 2011). Interestingly, in humans, migrating neuroblasts take a completely different route than in rodents and migrate into the SVZ-adjacent striatum, a migratory pathway that has not been observed in the injured rodent brain (Ernst et al., 2014). These findings are in line with other studies on non-human primates that have also shown that SVZ-derived adult-born neurons are present in the striatum (Bédard et al., 2002a, 2006). The recruitment of endogenous SVZ-derived neuroblasts in the brain of stroke patients (Jin et al., 2006; Macas et al., 2006) and in Huntington disease (Curtis et al., 2003) is associated with increased SVZ neurogenesis (Curtis et al., 2003, 2005; Martí-Fàbregas et al., 2010). Below we discuss the evidence for neuronal recruitment in stroke and some neurodegenerative disorders.

### Stroke

Stroke, which is induced by a poor blood supply to the brain, is one of the leading causes of death and disability in adulthood. In rodents, after middle cerebral artery occlusion, SVZ neurogenesis is enhanced, and neuroblasts are found at the site of the ischemic damage. Interestingly, these new neurons are generated up to a few months after the ischemic event (Thored et al., 2006). In human post-stroke brains, newborn neuron (Ki67^+^, DCX^+^, βtubulin^+^ and TUC-4^+^) clusters around blood vessels are found in the ischemic penumbra (Jin et al., 2006). Ischemia of the forebrain also leads to pyramidal hippocampal loss (Smith et al., 1984) and induces NPC proliferation in the dentate gyrus (Liu et al., 1998; Kee et al., 2001; Sharp et al., 2002). The new neurons in the ischemic brain are able to integrate the pre-existing network and form functional synapses (Yamashita et al., 2006; Hou et al., 2008).

The new neurons migrate through a *de novo* migratory route by re-activating some of the mechanisms that orchestrate neuronal migration in the uninjured RMS. As in the RMS, the migratory cells keep their chain structure (Zhang et al., 2004) and migrate along blood vessels (Yamashita et al., 2006; Grade et al., 2013). Some of the blood vessels in the ischemic striatum also start to express BDNF, which is used by neuroblasts to migrate from the SVZ-RMS toward the ischemic site (Grade et al., 2013). Treatment of stroke-induced mice with a small molecule targeting TrkB receptors revealed that neurogenesis increases in the SVZ and that there are greater numbers of newly generated neurons close to the stroke region (Han et al., 2012). This is associated with improved recovery in some motor behavior tasks (Han et al., 2012). Hydrogel delivery of BDNF directly to the injury site leads to both the recruitment and the maturation of neuroblasts in the injury site (Cook et al., 2017). It has been also shown that EGFR signaling increases in NSCs and progenitors following neonatal hypoxia/ischemia (Alagappan et al., 2009). The infusion of EGF and FGF-2 in the lateral ventricle of mice with CA1 pyramidal neuron loss increases neurogenesis and leads to the migration of neuronal progenitors into the CA1 (Nakatomi et al., 2002). Glial cells also play an important role in the regulation of migration. Glial cells surrounding the stroke area release SDF-1α, a chemokine that act on CXCR4 receptors expressed by migrating neuroblasts to attract cells toward the ischemic site (Robin et al., 2006; Thored et al., 2006). Migrating neuroblasts use the Slit1-Robo2 pathway to repel astrocytic processes and maintain a migratory route in order to pass through reactive astrocytes and infiltrate the injury site in the post-stroke brain (Kaneko et al., 2018). The inhibition of MMPs by GM6001, a broad spectrum inhibitor, decreases the migration of neuroblasts toward the ischemic site, indicated that MMPs also play an important role in controlling the migratory environment of neuroblasts (Lee et al., 2006).

Bcl-2 is a key factor for the survival of new neurons in the normal brain. Its overexpression leads to an increase in the survival of immature and mature neurons following ischemia in the hippocampus, striatum, and cortex (Sasaki et al., 2006; Zhang et al., 2006; Lei et al., 2012). Bcl-2 is highly expressed in the SVZ of humans (Bernier et al., 2000) and in the RMS of non-human primates (Bédard et al., 2002b). Bcl-2 phosphorylation promotes the activation of autophagy (Qi et al., 2015), which is involved in neuroblast migration (Bressan et al., 2020). Chemical manipulation of the autophagy pathway by targeting the interaction between Bcl-2 and beclin might be an interesting approach to increase autophagy activation. Candidate molecules have been already identified (Chiang et al., 2018). This approach could be used to modulate autophagy in migrating cells without inducing cell death. Bcl-2 family members also stimulate a large variety of signaling pathways involved in migration, MMP expression, PI3K and MAPK signaling, mitochondrial respiration, and ROS production (Um, 2016). The role of autophagy in stroke-induced progenitor migration requires further investigation as this process could act as both a pro-survival and pro-migratory pathway and as a pro-apoptotic pathway.

Other pharmacological compounds with autophagic effects have been studied for their positive effect on neurogenesis and cell migration. Metformin, an FDA-approved drug, promotes NSC proliferation and recruitment. Metformin injections in a mouse model of neonatal hypoxia/ischemia increased NSC proliferation in the SVZ and rescued motor deficits (Dadwal et al., 2015; Ruddy et al., 2019). Interestingly, the impact of metformin seems to be age- and sex-dependent in mice (Ruddy et al., 2019). In a mouse model of MCAO, metformin injections increase AMPK activation and promote neurogenesis and angiogenesis (Jin et al., 2014; Liu et al., 2014; Venna et al., 2014). However, it is important to note that AMPK activation during stroke may be detrimental (Li et al., 2007, 2011). As metformin interferes with cellular energy homeostasis, it would be interesting to investigate in detail how this drug acts on cell migration.

### Neurodegenerative Disorders

#### Huntington Disease (HD)

HD is a neurodegenerative disorder caused by a mutation in the huntingtin gene that leads to a polyglutamine-expansion of the protein, resulting in toxicity for striatal and prefrontal cortex neurons (Sassone et al., 2018). Neurogenesis increases in the HD human brain, with levels of PCNA^+^ cells paralleling the severity of HD (Curtis et al., 2003, 2005). Interestingly, in HD patients, hippocampal neurogenesis does not seem to be affected (Low et al., 2011). Striatal injections of quinolinic acid that mimic the neuropathology of HD increase neurogenesis in the rodent SVZ and lead to the appearance of newly generated neuroblasts and neurons in the lesioned striatum (Tattersfield et al., 2004). Quinolinic acid increases TNFα chemokine production in astrocytes (Guillemin et al., 2003), which promotes SVZ-derived neuroblast migration (Katakowski et al., 2007). Interestingly, immunohistological and carbon-14 dating studies of post-mortem human tissue have shown that SVZ-derived progenitors migrate into the striatum instead of the OB, indicating that the migratory trajectories of immature neurons in the human brain are redirected (Ernst et al., 2014). However, in HD patients, these adult-born striatal neurons become depleted over time (Ernst et al., 2014). Strategies aimed at improving their functional integration may help recovery and neuronal network restoration.

#### Parkinson's Disease (PD)

PD is induced by the degeneration of substantia nigra dopaminergic neurons and is associated with major motor disabilities. Interestingly, both mouse and human post-mortem studies have shown that the degeneration of dopaminergic neurons in PD leads to decreased proliferation of neuronal precursors (Hoglinger et al., 2004; Winner et al., 2009; Marxreiter et al., 2013). This hampers the regenerative response of endogenous neuronal progenitors and affects the integration and longevity of grafted cells, leading to deficient cell replacement therapies. Interestingly, in the 6-OHDA model of PD, the infusion of TGFα into the striatum increased SVZ neurogenesis and attracted neuroblasts to the infusion site (Fallon et al., 2000). TGFα acts on EGFR to potentiate proliferation in the SVZ and migration (Tropepe et al., 1997; Burrows et al., 2000; Junier, 2000). The migration of grafted hNSCs, the differentiation of a small number of cells into TH^+^ cells, and behavioral functional recovery have been documented in an MPTP primate model of PD (Redmond et al., 2007; Bjugstad et al., 2008). However, a large majority of grafted hNSC-derived cells along the nigrostriatal pathway exhibit glial markers such neurotrophic factor GDNF (Ourednik et al., 2002; Redmond et al., 2007; Bjugstad et al., 2008). As such, it is difficult to attribute recovery to the integration of the new neurons. The functional recovery observed in these studies could be due to a protective effect induced by hNPC graft cells (Ourednik et al., 2002; Redmond et al., 2007). This rescue could be due to GDNF expression by hNPC graft cells as GDNF protects against neuronal loss in animal PD models (Choi-Lundberg et al., 1997; Kordower et al., 2000; Redmond et al., 2007).

#### Alzheimer's Disease (AD)

While immature DCX^+^ cells are still present in the dentate gyrus of aged human brains, recent studies have shown that decreased DCX^+^ cell density in the DG of AD patients is correlated with the severity of the cognitive disorders (Moreno-Jimenez et al., 2019; Tobin et al., 2019). The decrease in the number of DCX^+^ cells is accompanied by a delay in cell maturation at the early stage of the disease before the appearance of ß-amyloid plaque (Moreno-Jimenez et al., 2019). A correlation analysis revealed that there is an association between the cognitive score and the number of DCX^+^/PCNA^+^ cells (Tobin et al., 2019). However, DCX^+^ cells can be still found in advanced AD cases (Braak stage V and VI) (Moreno-Jimenez et al., 2019). The decrease in the maturation of newborn neurons seems more likely associated with the importance of the cognitive dysfunction (Tobin et al., 2019). The decrease in neurogenesis could be due to changes in the expression of bone morphogenetic proteins (BMPs), a family of proteins involved in adult neurogenesis (Colak et al., 2008), as the decrease in neurogenesis is associated with an increase in BMP6 and BMP4 gene expression in the AD human brain and the AD mouse model (Li et al., 2008; Crews et al., 2010).

AD and PD are more frequent in aged patients in whom neurogenesis has declined significantly. The migration of neuronal precursors is also hampered in these disorders. Furthermore, the degeneration of dopaminergic neurons in PD leads to a further decline in neurogenesis. These neurodegenerative disorders are also associated with a large number of mutations in autophagy-related genes (Levine and Kroemer, 2019) that may, in addition to their involvement in neurodegeneration, affect neuronal production and migration. All these limitations are important to take into consideration when developing new strategies for boosting neuronal migration and regeneration in these neurodegenerative disorders.

## Conclusions

Physiological and pathological cell migration is a complex mechanism driven by several extracellular factors (molecular, scaffolding, mechanical, and electrical) that need to be integrated at the cellular level. These factors modulate cell behavior and trigger intracellular processes affecting cell migration. Cytoskeleton remodeling, mitochondria dynamics, ATP production, autophagy, and Ca^2+^-dependent pathways are important intracellular mechanisms located at the crossroads of many processes and on which a plethora of extracellular cues impinge to modulate cell migration. In pathologic conditions such as stroke, HD, and PD, neuronal precursors can migrate toward the damaged brain regions. While this endogenous reparative response is not sufficient to lead to functional recovery, its presence raises a lot of hope for developing new therapeutic strategies based on the use of endogenous or grafted NPCs. However, further progress in our understanding of the mechanisms regulating neuronal migration under homeostatic and pathological conditions as well as the differentiation, survival and functional integration of neuronal precursors is required in order to develop therapies that promote the regeneration of neuronal networks in diseased brain regions.

## Author Contributions

CB and AS wrote the review article. Both authors contributed to the article and approved the submitted version.

## Conflict of Interest

The authors declare that the research was conducted in the absence of any commercial or financial relationships that could be construed as a potential conflict of interest.
